# Laparoscopic Spleen-Preserving Splenic Hilar Lymphadenectomy Performed by Following the Perigastric Fascias and the Intrafascial Space for Advanced Upper-Third Gastric Cancer

**DOI:** 10.1371/journal.pone.0090345

**Published:** 2014-03-06

**Authors:** Chang-Ming Huang, Qi-Yue Chen, Jian-Xian Lin, Chao-Hui Zheng, Ping Li, Jian-Wei Xie, Jia-Bin Wang, Jun Lu

**Affiliations:** Department of Gastric Surgery, Fujian Medical University Union Hospital, Fuzhou, Fujian Province, China; Roswell Park Cancer Institute, United States of America

## Abstract

**Background:**

Laparoscopic spleen-preserving Splenic hilar lymphadenectomy (LSPL) is required in laparoscopy-assisted total gastrectomy for advanced proximal gastric cancer. However, it is considerably difficult and risk in clinical practice. Thus, we explore the application of LSPL performed by following the perigastric fascias and the intrafascial space in D2 radical gastrectomy for advanced upper-third gastric cancer.

**Methods:**

From July 2010 to December 2012, 109 patients with T2–3 upper-third gastric cancer underwent LSPL. Of these patients, 55 underwent classic LSPL (classic group), and the remaining 54 patients underwent LSPL performed by following the fascias and intrafascial space (fascia group). Clinicopathologic characteristics and intraoperative and postoperative variables were compared between the two groups.

**Results:**

There were no significant differences in clinicopathological characteristics between the two groups (P>0.05). All of the operations were successful without conversion to laparotomy. The operation time, mean splenic hilar lymph node (LN) dissection time, mean total blood loss and mean blood loss from splenic hilar LN dissection were significantly lower in the fascia group than in the classic group (P<0.05), whereas the times to first flatus, fluid diet and soft diet and the duration of hospital stay were similar in both groups. The mean number of harvested LNs (No. 10 and No. 11d) was slightly higher in the fascia group, but the difference was not significant. No significant difference in morbidity was found between the fascia group and the classic group (9.3% vs.10.9%, P>0.05). At a median follow-up of 12 months(range 5 to 35 months), none of the patients had died or experienced recurrent or metastatic disease.

**Conclusion:**

LSPL performed by following the fascias and intrafascial space is an optimal and safe technique based on anatomical logic, and it reduces the difficulties associated with LSPL, making it easier to master and allowing its widespread adoption.

## Introduction

The lymph nodes (LNs) in the splenic hilar area, including LNs along the distal splenic vessels (No. 11d) and the splenic hilum (No. 10), should be removed for a normative D2 LN dissection during total gastrectomy for advanced upper gastric cancer [1]. Although pancreatosplenectomy has been advocated for the complete removal of LNs in the splenic hilar area [2], [3], it is only performed in cases with direct tumor extension to the distal pancreas and spleen or with definite LN metastasis at the splenic hilum due to the high incidence of associated postoperative complications and mortality [4]. Moreover, patients who undergo pancreas- and spleen-preserving splenic hilar lymphadenectomy have lower morbidity and mortality rates than those who are subjected to distal pancreatectomy and splenectomy, with similar survival and recurrence rates. Therefore, spleen-preserving splenic hilar lymphadenectomy is now widely used in total gastrectomy with D2 lymph node dissection [5]–[7].

However, due to the complexity of the splenic hilar vessels, the anatomical variation, and the narrow and deep space at the splenic hilum, it is a difficult and risky operation, even in open surgery. In traditional open surgery, the surgeon can fully free the tail of the pancreas and the spleen through mobilization of the spleen in vivo to thoroughly dissect the LNs in the splenic hilar area; however, the same method cannot be used during laparoscopic operations. At the same time, because of the narrow laparoscopic vision and the lack of overall anatomical view during laparoscopic spleen-preserving splenic hilar lymphadenectomy (LSPL), surgeons (particularly beginners) easily lose their sense of position and direction, as they lack a fixed reference point, and enter the wrong anatomical layers, causing iatrogenic injury app:ds:substance. Therefore, it is important to identify methods that can improve the safety of the procedure, reduce the rate of associated iatrogenic injury and achieve the same radical effect as open surgery during LSPL.

Studies of laparoscopic total mesorectal excision for rectal cancer have shown that choosing the appropriate surgical area according to the potential anatomic space around the rectum can improve the operation efficiency and reduce injury and that it is more in line with the principles of “en-bloc” resection [8]–[10]. Based on special morphological characteristics, the anatomical distribution, and the relationship of the perigastric fascias and the intrafascial space during laparoscopic surgery and embryological development, laparoscopic radical gastrectomy for gastric cancer can be conducted following those fascias and the intrafascial space. Therefore, we describe LSPL performed by following the perigastric fascias and the intrafascial space and retrospectively compare the clinical data of upper gastric cancer patients who underwent this procedure with those of patients who underwent classic LSPL to investigate its safety and feasibility.

## Materials and Methods

### Embryological and anatomical background

At 4 weeks of gestation, the stomach is located in the midline and suspended by mesenteries composed of double layers of peritoneum. The peritoneum between the stomach and posterior body wall is known as the dorsal mesogastrium (DM). The spleen, pancreas and celiac branches originate from the space between the two layers of the DM. With the progression of embryo development, the stomach rotates from the sagittal to the coronal position, and the DM folds and expands to the lower left corner and forms two layers (anterior and posterior), each with two leaves. It gradually forms a large sac on the backside of the stomach, which is called the omental bursa. The DM is divided into two parts due to the presence of the spleen. The part between the spleen and stomach is called the gastrosplenic ligament (GSL), which provides a pathway for the short gastric and left gastroepiploic vessels (LGEVs). The section between the spleen and left kidney is known as the splenorenal ligament (SRL), which acts as a pathway for the splenic vessels and their branches. The anterior layer of the posterior leaf of the DM, which encompasses the pancreas, evolves into the anterior pancreatic fascia (APF) in front of the pancreas, whereas the posterior layer of the posterior leaf of the DM is attached to the posterior abdominal wall, which fuse together to form the posterior pancreatic fascia (PPF). At 3months of gestation, as the omental bursa crosses the transverse colon, the posterior leaf is fused with the primitive transverse mesocolon and degenerates to form the anterior lobe of the transverse mesocolon (ATM). After rotation of the embryonic foregut, the mesentery fuses with the mesentery, organs and abdominal wall. They close and stick together to form a potentially widely distributed anatomical plane that is full of loose connective tissue, called the fusion fascia [11]. The fusion fascia is a natural avascular zone containing loose connective tissue, and it has a very distinct appearance from the mesentery, which is rich in fat tissue. Hence,as a surgical plane, it can be used to easily guide the direction of separation [12].

**Figure 1:**
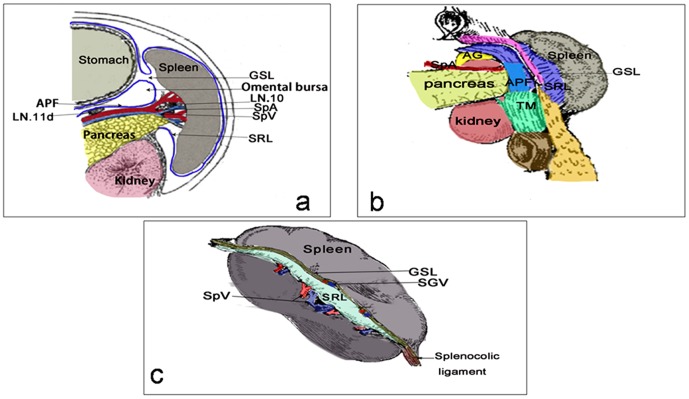
Schematic drawing of the fascias and the vascular anatomy around the splenic hilum. a. The anatomic relationships between the APF, GSL and SRL, which are all derived from the DM. b. The anatomic relationships between the connected fascias around the splenic hilum. c. The anatomic relationships between the GSL, the SRL, the terminal branch of the splenic vessels and the SGVs. APF, Anterior pancreatic fascia; SRL: Splenorenal ligament; GSL, Gastrosplenicligament; TM, Transverse mesocolon; AG, Adrenal gland; SGVs,Short gastric vessels.

The stomach, spleen and pancreas and their vasculatures are enclosed by the DM during the embryonic developmental stages. Although the DM evolves into different structures or fuses with adjacent structures due to the rotation of the foregut during gestation, the above-mentioned organs are still enclosed by this huge, extensively relative framework. Therefore, the fascias around the splenic hilum, including the GSL, SRL, pancreatic fascia, ATM and greater omentum, all evolve from the DM during gestation. Anatomically, they are connected to each other, and the intrafascial spaces between them are also mutually linked, whereas the vascular and lymphatic systems, which play primary roles in nutriment and support, must traverse the potential space formed by this double-layer fascia, regardless of variability or the existence of individual differences. LSPL performed by following the perigastric fascias and the intrafascial space is an operative technique that can be used to conduct a high-efficiency, sequential and safe splenic hilar lymphadenectomy under laparoscopic view (Fig. 1).

### Patients

Between July 2010 and December 2012, 109 patients with T2–3 upper-third gastric cancer underwent LSPL at Fujian Medical University Union Hospital. Of these patients, 55 underwent classic LSPL (referring to the procedure described in the literature [13]) before May 2012 (classic group), and the remaining 54 patients underwent LSPL following the fascias and intrafascial space after May 2012 (fascia group) with the same perioperative management. Clinicopathologic characteristics and intraoperative and postoperative variables were compared between the two groups.

Nodal material was separately dissected from the en-bloc specimen at the end of the procedure by the surgeons, and the remaining nodes were identified and retrieved by specialized pathologists from formalin-fixed surgical specimens without using any specific technique to increase the node-retrieval rate. The LNs of the stomach are defined and given station numbers according to the 3rd English edition of the Japanese classification of gastric carcinoma [14]. Staging was performed according to the 7th edition of the UICC tumor, node, and metastasis (TNM) classification [15].

The inclusion criteria were as follows: histologically confirmed adenocarcinoma of the stomach and upper advanced gastric cancer obtained during a comprehensive evaluation, including preoperative endoscopy, endoscopic ultrasound and abdominal computed tomography (CT); the depth of tumor invasion was T2–T3; no evidence of distant metastasis or para-aortic lymph node involvement by preoperative examination; and LSPL with curative R0 according to the pathological diagnosis after the operation. The exclusion criteria were as follows: the depth of tumor invasion was T1 or T4; intraoperative evidence of peritoneal dissemination or distant metastasis; observation of an extremely large tumor, enlargement or integration of the splenic hilar LNs during the operation; and incomplete pathological data. All of the procedures were performed after obtaining informed consent following the explanation of the surgical and oncologic risks.

### Ethics Statement

Ethics committee of Fujian union hospital approved this retrospective study. Written consent was given by the patients for their information to be stored in the hospital database and used for research.

### Surgical Procedures

**Figure 2:**
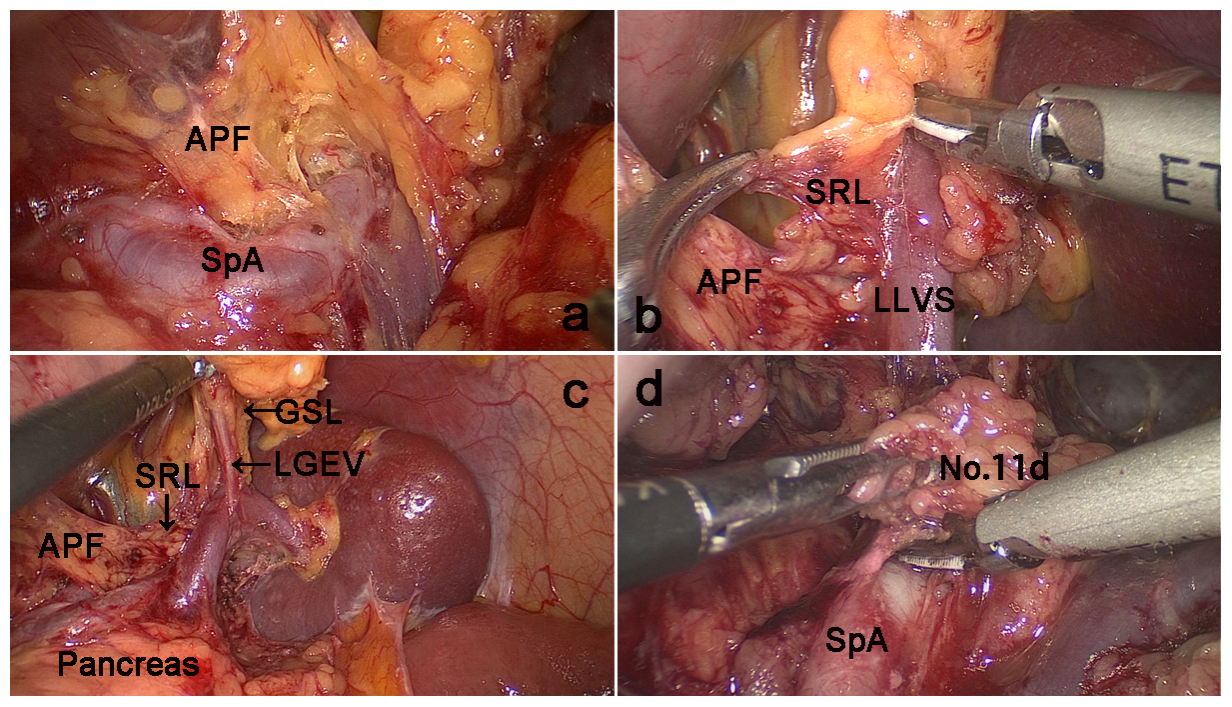
Dissection of the No. a. The APF was completely lifted cephalad to fully expose the superior border of the pancreas and enter the retropancreatic space (RPS). b.The lower lobar vessels of the spleen (LLVSs) were exposed between the two layers of the SRL by following the RPS c. The left gastroepiploic vessels were exposed by following the fascias. d. Dissection of the No. 11d LN was performed by following the intrafascial space. APF: Anterior pancreatic fascia; SRL, Splenorenal ligament; LLVSs, Lower lobar vessels of spleen; GSL, Gastrosplenicligament; LGEVs, Left gastroepiploic vessels; SPA, Splenic artery.

All of the operations were performed by the same group of gastric surgeons who had previously completed more than 500 cases of laparoscopic radical gastrectomy for gastric cancer. All of the surgeons used Rou-en-Y esophagus jejunum anastomosis to reconstruct the digestive tract. The procedure performed in the classic group is referenced in the literature [13]. In brief, we performed LSPL following the route of splenic vessels in the classic group, while we conducted the procedure following the orientation of perigastric fascias and the intrafascial space in the fascial group. We performed surgery in the fascia group using the following sequence: the space between the two leaves of the transverse mesocolon to the retropancreatic space (RPS) to the space between the SRL layers to the RPS to the space between the SRL to the space between the layers of the GSL. The detailed operation steps were as follows:

1. Position: The patient was placed in the reverse Trendelenburg position with their head elevated approximately 15 to 20 degrees and tilted left-side up approximately 20 to 30 degrees. The surgeon stood between the patient's legs, with the assistant and camera operator both on the patient's right side.
2. The assistant used their left hand to pull the GSL and their right hand to assist with the manipulation of the surgeon and maintain proper tension. First, the surgeon separated toward the inferior margin of the pancreatic tail along the fused intrafascial space between the ATM and the posterior lobe of the transverse mesocolon (PTM) using a harmonic scalpel. The separation was continued toward the back of the APF following the orientation of the fascias. Next, the APF was peeled toward the superior border of the pancreatic tail along the direction of the pancreas, closing toward the anterior inherent fascia of the pancreas. Then, the peeled ATM and APF were completely lifted cephalad to fully expose the superior border of the pancreas and enter the RPS (Fig. 2a).

The assistant pulled the fundus and body of the stomach to the upper right and tensed the GSL, while the surgeon gently pressed the body and tail of the pancreas to the lower left to expose the splenic hilum. In front of the pancreatic tail, the surgeon continued to follow the direction of the fascia to peel the APF and enter the space between the two layers of the SRL through the RPS. In general, this space was gradually expanded from the pancreatic tail to the splenic hilum. The lower lobar vessels of the spleen (LLVSs) or lower pole vessels of the spleen could then be exposed by following this space (Fig. 2b).(Next, within the two layers of the SRL, the surgeon used an ultrasonic scalpel to cut the surface of the lymphatic fatty tissue around the vessels to expose the LGEVs and divide them at the root (Fig. 2c).

**Figure 3:**
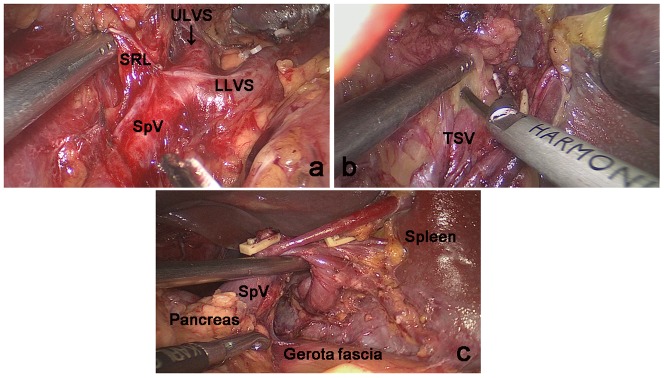
Dissection of the splenic hilar LNs was performed by following the perigastric fascias and the intrafascial space. a. The upper lobar vessels of the spleen (ULVSs) were exposed by following the space between the two layers of the SRL. b. The terminal branches of the splenic vessels were completely skeletonized by following the intrafascial space.c. Dissection of the adipose tissue around the lymph nodes behind the splenic vessels in front of Gerota's fascia. SRL: Splenorenal ligament; ULVSs ,Upper lobar vessels of spleen; LLVSs,Lower lobar vessels of spleen; SpVs, Splenic vessels; TSV, The terminal branch of splenic vessels

**Figure 4:**
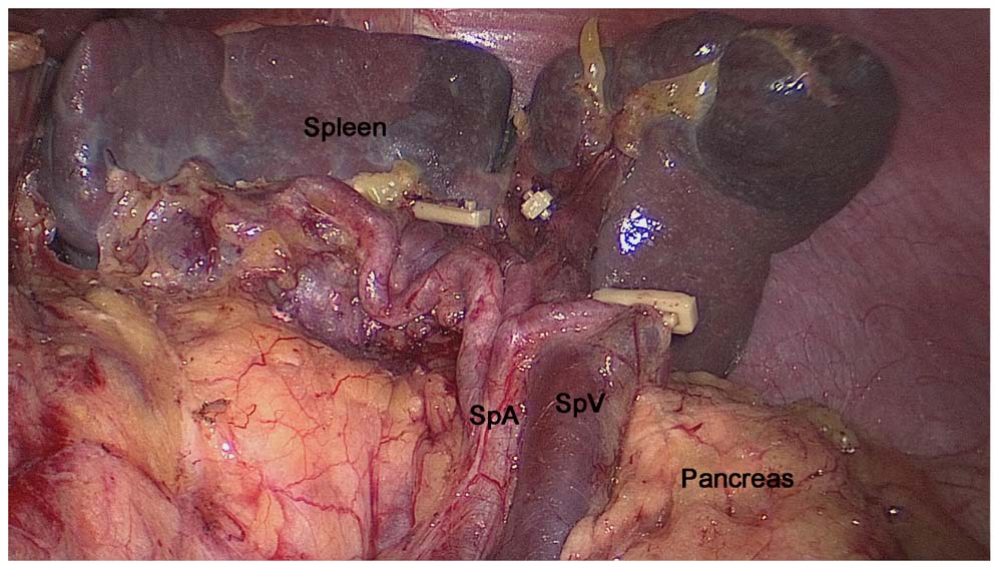
Intraoperative view after splenic hilar lymphadenectomy. SPA, splenic artery; SPV, splenic vein.

1. The space between the SRL layers, where the roots of the LGEVs lay, was used as the dissection plane to completely expose the LLVSs. During the dissection process, one or two branches of the short gastric vessels (SGVs) arising from the LLVSs and entering the GSL were skeletonized and divided at their roots within the SRL. Next, the assistant put the free omentum between the liver and the stomach and continually pulled the posterior wall of the fundus and body of the stomach to the upper right. The surgeon gently pressed the pancreas to fully reveal the RPS and the space inside the SRL. Then, the surgeon tracked the termini of the splenic vessels along the completely vascularized LLVSs within the space inside the SRL. Next, the surgeon carefully dissected the fatty lymphatic tissue around the splenic vessels (No. 11d) along the latent anatomic spaces on the surface of the splenic vessels (Fig. 2d).
2. The surgeon then reentered the space between the SRL layers following the RPS where the splenic vessels lay and opened the SRL in the splenic hilar area to expose the upper lobar vessels of the spleen (ULVSs) and the middle lobar vessels of the spleen (MLVSs). The assistant then gently pulled up the fatty lymphatic tissue at the surface of the terminal branches of the splenic vessels within the SRL and kept it under tension. The surgeon used the non-functional face of the ultrasonic scalpel to cut the surface of the terminal branches of the splenic vessels to completely skeletonize the vessels in the splenic hilum with a meticulous sharp or blunt dissection (Fig. 3b). Next, the fatty connective tissue, including the LNs around the splenic hilum (No. 10), was completely removed. Using this approach, the surgeon could always maintain the correct surgical plane and a clear understanding of the layers in the operative field. During the dissection process, two or three branches of the SGVs arose from terminal branches of the splenic vessels and entered the GSL, and they were also gradually skeletonized and divided at their roots in the SRL.Next, the portions of the SRL and GSL in the splenic hilar area were completely removed.
3. The assistant ventrally lifted the termini of the splenic vessels using atraumatic grasping forceps. The surgeon then dissected the adipose tissue surrounding the lymph nodes behind the splenic vessels in front of Gerota's fascia. Attention is required during this step so that the separation plane does not exceed Gerota's fascia, which may damage the kidney, adrenal gland and related vessels or nerves behind it (Fig. 3c). At this point, the splenic hilar lymphadenectomy is complete (Fig. 4).

### Statistical analysis

All statistical analyses were performed using the statistical program SPSS 18.0. Data were reported as the mean ±SD and compared using the chi-square test, Fisher's exact test or the unpaired Student's *t*-test, as appropriate. *P*<0.05 was considered statistically significant.

## Results

### 1. Patient clinicopathologic characteristics

The clinicopathologic characteristics of the 109 patients are presented in Table 1. The series included 87 men and 22 women with a mean age of 61.4 years (range 24 to 80 years). Age, gender, comorbidity, American Society of Anesthesiologists (ASA) score, body mass index (BMI), tumor size, tumor depth,lymph node status (N stage), TNM stage and histologic type did not differ between the two groups (*P*>0.05 each).

**Table 1:** Comparison of clinicopathological characteristics between the fascia group and the classic group.

Characteristics	Classic group(n = 55)	Fascia group(n = 54)	*P* value
Sex			0.316
Female	9	13	
Male	46	41	
Age(years)	61.2±11.2	61.6±9.1	0.863
ASA score			0.484
I	41	33	
II	12	17	
III	2	4	
BMI(kg/m^2^)	22.1±3.2	22.0±2.6	0.715
Tumor size(cm)	5.4±1.9	5.4±2.9	0.955
Tumor depth			0.637
T2	18	20	
T3	37	34	
pN stage			0.949
N0	16	16	
N1	10	11	
N2	11	12	
N3	18	15	
TNM stage			0.364
IB	7	4	
IIA	15	10	
IIB	5	8	
IIIA	15	12	
IIIB	13	20	
Histologic type			0.776
Differentiated	29	27	
Undifferentiated	26	27	

COPD, chronic obstructive pulmonary disease; CHD,coronary heart disease;CVD,cerebrovascular disease; ASA,American Society of Anesthesiologists; BMI, body mass index;TNM, tumor node metastasis staging; P-values are for comparisons between the fascia group and the classic group.

### 2. Intraoperative and postoperative characteristics

All of the 109 operations were successful. No patient required conversion to laparotomy, and none required splenectomy due to intraoperative injury to the splenic blood vessels or the spleen itself. The operation time, mean splenic hilar LN dissection time (from exposing the termini of the splenic vessels to the end of the splenic hilar lymphadenectomy), mean total blood loss and mean blood loss due to splenic hilar LN dissection were significantly lower in the fascia group than in the classic group (P<0.05 each) without intraoperative or postoperative blood transfusion. By contrast, the times to first flatus, fluid diet and soft diet and the duration of hospital stay were similar between the two groups (*P*>0.05 each) (Table 2).

**Table 2:** Intraoperative and postoperative characteristics.

Variables	Classic group(n = 55)	Fascia group(n = 54)	P value
Operation time(min)	176.6±33.7	162.3±22.8	0.011
SLNs dissection time (min)	30.2±12.1	18.5±4.7	<0.001
Total blood loss(ml)	55.1±20.0	41.8±15.9	<0.001
SLNs dissection blood loss (ml)	22.5±9.7	7.3±5.5	<0.001
Time to first flatus(d)	4.0±1.1	3.9±0.8	0.470
Time to fluid diet (d)	4.4±1.0	4.4±0.9	0.949
Time to soft diet (d)	7.5±1.9	7.1±0.8	0.172
Hospital stay (d)	10.8±2.9	10.3±1.5	0.218

SLNs, splenic hilar lymph nodes; P-values are for comparisonsbetween the fascia group and the classic group.

### 3.LN dissection

The mean number of total harvested LNs, the lymph node metastasis rate and the ratios of metastatic LNs (No. 11d and No. 10) did not significantly differ between the two groups (P>0.05 each). The mean numbers of harvested No. 10 and No. 11d LNs were slightly higher in the fascia group, but the difference was not significant (P>0.05 each) (Table 3).

**Table 3:** Lymph node dissection results for the two groups.

Variables	Classic group (n = 55)	Fascia group (n = 54)	P value
Mean total retrieved LNs	39.6±12.1	40.3±13.1	0.768
Mean retrieved NO. 10 LNs	2.8±2.1	3.0±2.7	0.607
NO. 10 LMR	5.5% (3/55)	7.4% (4/54)	0.949
NO. 10 RML	7.2% (11/152)	4.3% (7/162)	0.267
Mean retrieved NO. 11d LNs NO.11d	2.3±1.7	2.7±2.3	0.299
NO. 11d LMR	12.7% (7/55)	18.5% (10/54)	0.405
NO. 11d RML	7.6% (9/119)	16.4% (24/146)	0.085

LNs, l**ymph nodes**; LMR, lymph nodes metastasis rate; RML, the ratios of metastatic lymph nodes.

### 4. Morbidity and mortality

The overall postoperative morbidity was 10.1% (11/109). The postoperative complications did not differ between the fascia group and the classic group (9.3% [5/54] vs.10.9% [6/55], P>0.05). There were three cases of pulmonary infection, one pancreatic fistula, one chylous fistula and one abdominal infection in the classic group, whereas there were two cases of pulmonary infection, one anastomotic leakage, one inflammatory intestinal obstruction and one abdominal infection in the fascia group. All of these postoperative complications were successfully treated by conservative methods, and none of these patients required a second operation. No patient experienced a postoperative splenic infarction or hemorrhage of, or injury to, the splenic veins. The 30-day mortality rate in the total patient population was 0%.

### 5. Postoperative follow-up

Follow-up was carried out through June 2013.All of the patients were followed for a median of 12 months (range, 5–35 months). None of these patients died or experienced tumor recurrence or metastasis during follow-up.

## Discussion

D2 lymphadenectomy, including the removal of the No. 11d and No. 10 LNs, has become the standard surgical procedure for patients with curable upper gastric cancer [16], [17]. With the improvement of surgical techniques and the renewed concept of organ preservation, spleen-preserving splenic hilar lymphadenectomy has been widely accepted and applied by many surgeons in open D2 radical lymphadenectomy for upper gastric cancer. In recent years, as the safety, feasibility and short-term and long-term results of laparoscopic radical lymphadenectomy for gastric cancer have been gradually confirmed [18]–[21], few surgeons have carried out LSPL [22]–[24]. However, substantial differences in the operation time, intraoperative bleeding volume, postoperative complications and residual tumor rate have been observed among surgeons. These differences were particularly observed in beginners due to the complex technique used during free stomach or splenic hilar lymphadenectomy, during which intricate operative techniques and an intricate operating plane are required. Therefore, this technology has not been widely popularized in clinical practice. Some urgent problems remain unresolved. The operative field under a laparoscope lacks an overall anatomical orientation and a sense of distance due to the narrow visual field and two-dimensional images that are provided; moreover, the land marks used for positioning are relatively smaller than those used in open surgery. Perhaps worse, the operation field moves between a number of anatomical levels and areas with intricate vascular networks. The entire operative area still lacks a good single surgical plane. As a result, surgeons easily become disoriented and enter the wrong anatomical layers, causing iatrogenic injury during the operation.Due to lack of understanding of the holistic concept of the embryological origins and anatomical distributions of the relative fascias, the scope of resection is generally unclear, leading to unsuccessful radical resection.

The appropriate method for localizing the operating field in a safe, efficient surgical plane with an optimal range of lymph node dissection that is in agreement with the radical principles of oncology must be determined to improve operation efficiency and reduce blood loss, and as a result, promote the development and application of LSPL.

Based on special morphological characteristics, anatomical distribution, the relationship of the perigastric fascial and intrafascial spaces during the previous laparoscopic surgery as well as their embryological development, we identified an LSPL method performed by following the fascias and the intrafascial spaces to treat gastric cancer. In this way, we attempted to identify an optimal surgical plane and operating range for LSPL. The pancreatic fascia, ATM, GSL and SRL evolve from the DM during embryological development. Although their anatomical morphologies have appreciable differences, they are connected to each other, and the intrafascial spaces between them are also mutually linked. Therefore, we first completely peeled the ATM and APF to enter the RPS and expose the LLVSs as well as the partial splenic vascular trunk. Next, the SRL and GSL were separated along the fascia to completely reveal all of the splenic vessels and their branches. The surgeon was then able to dissect the LNs along the distal splenic vessels and the splenic hilum along the latent anatomic spaces with great ease. The supporting vascular and lymphatic systems must travel through those intrafascial spaces, regardless of variability or individual differences [25]. Therefore, the space between two layers of the DM can be used as a surgical plane to guide the separation and standardize the operating range in LSPL. Moreover, with laparoscopic amplification and the superior effects of ultrasonic scalpels for cutting and hemostasis, the surgeon can more clearly visualize the perigastric fascia, intrafascial space, vasculature, nerves and other structures. Thereby, the splenic vessels and their branches can be comfortably exposed at different levels, and the meticulous procedure of the splenic hilar area lymphadenectomy can be smoothly and efficiently completed without unexpected hemorrhage or injury to the spleen or pancreas.Our data showed that the operation time, mean splenic hilar LN dissection time, mean total blood loss and mean blood loss due to splenic hilar LN dissection were significantly lower in the fascia group than in the classic group. Moreover, morbidity was also slightly lower in the fascia group, without abdominal organ injuries such as pancreatic fistula and chylous fistula. No patient required conversion to laparotomy, and none required splenectomy as a result of intraoperative injury to the splenic blood vessels or the spleen itself. Therefore, following the fascias and the intrafascial spaces during LSPL is an operative technique that can provide a safe and sequential anatomical plane for surgeons, and it can guide the operating direction throughout the entire process, including during vessel skeletonization and LN dissection. As a result, surgeons can reduce aimless exploration, shorten the operative time and decrease intraoperative bleeding. Therefore, performing LSPL along the fascias and the intrafascial spaces reduces the difficulty and risk of the operation, allowing it to be more easily learned and promoting its application and popularization in D2 radical gastrectomy for advanced upper gastric cancer.

The perigastric fascias and intrafascial space are derived from the DM, the ventral mesogastrium and the loose connective tissue, which are attached to each other. They therefore provide a predominant pathway for the systematic dissemination of malignant or inflammatory lesions. Simultaneously, the lymphatic system, including the LNs and lymphatic vessels, is located within the fascias along with vessels. Hence, the radical resection of gastric cancer is not merely performed by dissecting the related LNs; the corresponding fascias should also be removed to achieve an oncological “en bloc resection”. It is only in this manner that can we effectively prevent residual cancer caused by micrometastases and truly achieve a radical cure effect [12]. Laparoscopic amplification elaborately demonstrates the boundary between the perigastric fascias and the adjacent fascia, which allows surgeons to completely separate the fascias and the related lymphatic vessels contained within. Thus, this procedure appears to be more accordant with the radical principles of oncology. Our results revealed that the mean number of harvested LNs (No. 10 and No. 11d) was slightly higher in the fascia group and was equivalent to the number of LNs harvested by biopsy [26], which also reflects its advantage in oncologically radical treatment. Although the average BMI of both groups were about 22, we also can successfully performed the LSPL in some higher BMI patients using the strategy of following the perigastric fascias and the intrafascial space. However, more targeted clinical trials to evaluate its surgical safety and oncological efficacy for higher BMI patients are needed.

In conclusion, LSPL following the perigastric fascias and the intrafascial space, which is a novel design, is a safe, feasible and optimal technique based on anatomical logic. It reduces the difficulties associated with LSPL and allows it to be more easily mastered and promoted. As a result, LSPL can be more easily applied in D2 radical gastrectomy. However, to establish its curative effect for upper gastric cancer, multicenter, randomized, controlled trials evaluating long-term outcomes are necessary.
